# Darkness-induced effects on gene expression in *Cosmarium crenatum* (Zygnematophyceae) from a polar habitat

**DOI:** 10.1038/s41598-019-47041-7

**Published:** 2019-07-22

**Authors:** Florian Mundt, Dieter Hanelt, Lars Harms, Sandra Heinrich

**Affiliations:** 1 University of Hamburg, Institute of Plant Science and Microbiology, Aquatic Ecophysiology and Phycology, Hamburg, Germany; 2 Alfred-Wegener-Institute Helmholtz-Centre for Polar and Marine Research, Scientific Computing, Bremerhaven, Germany; 3 University of Hamburg, Institute of Plant Science and Microbiology, Molecular Plant Genetics, Hamburg, Germany

**Keywords:** Microbiology, Transcriptomics, Light responses

## Abstract

Light is a key environmental regulator in all photosynthetic organisms. Many studies focused on the physiologic response to changes in light availability of species from the Zygnematophyceae, but the impact of the absence of light and the molecular acclimation process on the other side have been poorly understood. Here we present transcriptomic analyses of *Cosmarium crenatum* from a polar habitat exposed to darkness. The algae were cultured in dark for one week; cell number and quantum yield of photosystem II (Fv/Fm) were monitored. Cell number was stable, but the Fv/Fm decreased in both groups, darkness-treated and control. Gene expression analysis revealed a strong repression of transcripts associated with photosynthesis, photorespiration and cell wall development. General carbohydrate and lipid metabolism were differentially regulated, but starch is shown to be the primary energy source in these conditions. Additionally, *C. crenatum* induced mRNA responsible for epigenetic modifications which may be a specific response to an adaption and acclimation to polar conditions. Our study sheds light on the molecular acclimation process to darkness and provides ecological implications for new perspectives in this specialized group of green algae.

## Introduction

Numerous studies showed that light is a key regulator of plant metabolism, development and a central factor for survival. Change of gene expression as reaction to alterations of light availability has been studied in model land plants since decades^1,2^. Plants fast-react to changes of environmental conditions in order to survive and therefore exhibit a complex remodeling of the transcriptome^3^. The acclimation to various abiotic conditions of photosynthetic organisms was key for the colonization of terrestrial habitats about 450 Mya^4^. Land plants evolved from an aquatic ancestor from the group of Charophyta, sharing fundamental phenotypic traits^5–7^. The genus *Cosmarium* forms the largest taxonomic entity within the Zygnematophyceae^8^, the closest living relatives to the Embryophyta^9,10^. This makes *Cosmarium* and other genera of the conjugating green algae useful organisms to study early land plant evolution^11^.

A broad physiological acclimation potential has been described for different *Cosmarium* species with origins ranging from the tropics to polar regions^12^. Furthermore, the physiological acclimation to certain environmental regimes points to the origin of the isolate^13^, but molecular information on the acclimation process is still missing. The species *Cosmarium crenatum* is distributed worldwide and has been discovered in e.g. Scotland^14^, the northwest territories in Canada^15^, New Zealand^16^ as well as the sub Antarctic Kerguelen islands^17^. Described as an essentially arctic-alpine species, *C. crenatum* is predominantly found under harsh climatic conditions of high altitude or latitude, but exhibits a great acclimation potential, and thus, may be encountered even in the lowlands^18^. In these wide-spread localities environmental factors like temperature and nutrients differ tremendously, but the availability of light may be the most challenging factor for a photoautotroph. Polar freshwater ponds are subjected to continuous winter darkness with ice covers persisting up to 9 months^19^. Under different climate change scenarios, a rise in temperature is projected to increase precipitation and cloud cover in high Arctic regions^20^ and, therefore, the ice-covered period may even be extended in the future. Photosynthetic primary production in polar regions is strongly influenced by underwater irradiance which highly depends on prolongation of snow cover in spring, latitudinal position and turbidity^21,22^. Water temperatures below 0 °C are ubiquitous in winter in Arctic and Subarctic regions, but also above 20 °C may be easily reached in shallow ponds in summer^23^. The physiological acclimation to naturally occurring abiotic stress scenarios of a number of charophyte green algae has been well studied^24^ and molecular acclimation studies in these non-model organisms are usually built around specific stressors e.g. desiccation^25,26^ or the influence of phytohormones^27^. Although many physiological studies were performed on temperature and light stress in these groups, different stressors are poorly understood and molecular information on the process of acclimation and comprehensive metabolic information is still missing.

This study aims to characterize the molecular response to long-term darkness-induced changes in gene expression in the non-model microalga *C. crenatum*. Furthermore, we established a comprehensive *de novo* transcriptome as a basis for future studies on the mechanisms involved in light and temperature acclimation. It is our key hypothesis that an extensive remodeling of the transcriptome is necessary for the alga for survival during elongated darkness as photosynthesis cannot be the energy source.

It is our long-term goal to understand the molecular basis of acclimation to environmental alterations like the influence of polar night. A RNA-seq approach was utilized to construct the transcriptome reference and to investigate differential gene expression. Physiological responses to darkness of one week were determined by cell growth and the maximum quantum yield of photosystem II and compared to gene expression changes.

## Material and Methods

### Algal material

Liquid cultures of a polar strain of *C. crenatum* Ralfs 1884 var. *boldtianum* Gutwinski (Microalgae and Zygnematophyceae Collection Hamburg (MZCH) 561), originating from Franz Josef Land (Russia), were cultured in a synthetic mineral Woods Hole (WH) medium^28^ at 12 °C and 100 µmol photons m^−2^ s^−1^ in a light:dark cycle of 16:8 h. The cultures were acclimated to culture medium and conditions at least 6 months before the experiment was carried out. The algae were pre-cultured in separate 1 L Erlenmeyer bottles filled with 800 mL medium and dispersed with atmospheric air. For gaining high cell concentrations, dense pre-cultures were disconnected from gasification and irradiated 1 h under standard conditions. Algal cells started to form dense conglomerates and rose to the upper surface layer. The algae were transferred with sterile 10 mL pipettes to clean glass bottles and examined prior with an inverted microscope to check vitality and purity of the culture.

### Experimental conditions and reference transcriptome RNA sampling

Cultures were transferred from standard growth conditions at 12 °C and 100 µmol photons m^−2^ s^−1^ to 2, 12, 22 and 32 (±1) °C for one week at 100 and 500 µmol photons m^−2^ s^−1^ (n = 8) in a light:dark cycle of 16:8 in a Multi-Cultivator MC 1000-OD (Photon Systems Instruments, Brno, Czech Republic). Additionally, algae were cultured at 2 °C in darkness for one week. Sampling was performed initially (1 h), short-term (16 h) and long-term (1 week), replicates n = 8. An external cooling aggregate was mounted to the MC for the treatments at 2 and 12 °C. Exposures to UV-radiation (UVR) were performed short-term (16 h) at all mentioned temperatures for high and low light (500 and 100 µmol photons m^−2^ s^−1^) exposure in a sun simulator (SonSi, iSiTECGmbH, Bremerhaven, Germany). For a solar-like spectrum including UVR (UVA 8.8 Wm^−2^, UVB 0.61 W m^−2^), the cultures were illuminated with a 400 W Metallogen lamp (Philips MSR 400 HR, Germany), as described in literature^29,30^. After exposure, samples from all treatments were collected. Additionally, samples from standard culture conditions and from one day, one week and one month old cultures (n = 3) were taken. Samples were centrifuged in 2 mL reaction tubes (Sarstedt, Nümbrecht, Germany) for 10 s, the liquid phase was discarded. The tubes were initially flash-frozen in liquid nitrogen and stored at −80 °C until RNA extraction.

### Physiology

Cell number was measured with a particle counter (Beckman Coulter GmbH, Krefeld, Germany) with a tube aperture of 100 µm. Chlorophyll fluorescence was determined *in vivo* with an Imaging Pulse Amplitude fluorometer (Imaging PAM, Heinz Walz GmbH, Effeltrich, Germany) and analyzed with the ImagingWin software (Heinz Walz GmbH, Effeltrich, Germany). Chlorophyll fluorescence was measured as Fv/Fm ((Fm/-Fo)/Fm), which is the optimal quantum yield indicating photosynthetic efficiency of dark adapted reaction centers, i.e. which amount of absorbed photons may be converted into electron transport. The maximum fluorescence (Fm) is the theoretical highest possible fluorescence where all reaction centers are closed, the minimum fluorescence (Fo) is the fluorescence when all reaction centers are open. (for further information see^31^). Prior to the measurements, the number of cells was adjusted to approximately 110,000 cells mL^−1^ with fresh culture medium ensuring comparable results of the photosynthetic activity and to avoid fluorescence reabsorption. Photosynthetic data and cell numbers were statistically analyzed with SPSS software version 23 (IBM, Armonk, USA). Based on homogeneity of variance tested with Levene statistic and normality testing with Lilliefors significance correction on Kolmogorov-Smirnov test, either an ANOVA or a non-parametric Friedman’s test was applied (*p* ≤ 0.05).

### RNA isolation and reference transcriptome sequencing

RNA extraction was performed with guanidine thiocyanate phenol chloroform, following the protocol for microalgae samples^32^. After RNA purity control with a NanoDrop 2000 spectrophotometer (Thermo Fisher Scientific, Waltham, MA, USA), all samples with high purity and quantity were assessed on a Fragment Analyzer (Advanced Analytical Technologies Inc., Heidelberg, Germany).

Two sequencing approaches were utilized, the first to generate a comprehensive *de novo* reference transcriptome and, the second to profile the gene expression after one week of darkness exposure. For the reference transcriptome generation, high quality samples with RNA quality number (RQN) above 6 (214 in total) of differentially treated algae from all experimental conditions were pooled in equal amounts to generate a normalized reference transcriptome. The pooling of various RNA samples from different treatments was conducted to ensure a high functional coverage of the reference transcriptome. Reference library sequencing was carried out with a genome sequencer Illumina HiSeq 2500 in rapid run 300 bp paired-end modus.

### Gene expression sequencing

The cDNA libraries for analysing differential gene expression in darkness were constructed in triplicates. Strand-specific cDNA libraries for analyzing differential gene expression were sequenced in HiSeq 2500 rapid run 100 bp single read modus. All library preparations and sequencing were carried out by GATC Biotech (Konstanz, Germany). The cleaned raw data were deposited in the European Nucleotide Archive (ENA) at the European Molecular Biological Laboratory–European Bioinformatics Institute under study accession number PRJEB30351.

### Transcriptome assembly and annotation

Quality of reads were controlled with FastQC^33^. Adapter sequences were removed with bbduk.sh, from the BBtools suite, version 36.38^34^ with following parameters: ktrim = r, k = 23, mink = 11, hdist = 1, tpe, tbo. Remaining sequences were searched for rRNA sequences by SortMeRNA version 2.1^35^ which were removed before further processing. To filter the sequences for the common Illumina spikein PhiX, bbduk.sh was used with a kmer size of 31 and a hdist of 1. A final quality trimming was performed with bbduk.sh using Q10 as minimum quality and 36 bases as the minimum length. The quality filtered reads were assembled de novo into transcripts yielding 141,711 transcripts using the Trinity assembler version 2.5.1^36^ with normalized max. read coverage of 100 and a minimum transcript length of 300 bp as parameters. To estimate the completeness of the reference transcriptome Benchmarking Universal Single-Copy Orthologs (BUSCO) was used with the plant datasets 1.1 and Embryophyta odb9 as databases^37^. The generated transcripts were functional annotated using the Trinotate annotation suite version 3.0.2 (https://trinotate.github.io/). Functional annotation included homology search against known sequence data (BLAST+/UniProtKB Swiss-Prot), protein domain identification (HMMER/PFAM) and retrieving data from additional databases (KEGG/COG/EggNOG). To assess the coverage of metabolic and regulatory pathways, the assigned KEGG, Kegg orthology (KO) numbers and COG were mapped to a KEGG^38–40^ pathway map (Supplementary Fig. S1).

The reference transcriptome of *C. crenatum* was compared in a reciprocal best hit analysis with the transcriptome of *Zygnema circumcarinatum* (Zygnematophyceae)^26^ and the coding sequences of the genome of *Marchantia polymorpha* (Embryophyta)^41^. Using Transdecoder version 3.0.0 (http://transdecoder.github.io), protein sequences were predicted for all three species, which were then used for a reciprocal blast search applying an e-value cut-off of 1e^−7^.

### Differential expression analysis

Short reads of each sample were aligned against the reference *de novo* transcriptome, using Bowtie2 (version 2.3.3). Low-count transcripts were filtered using a row sum cut-off (>10) on the generated count matrix. Relative abundances were estimated by RSEM (version 1.2.26) and genes were analysed for differential expression using the edgeR Bioconductor package^42^ with a standard level of *p* ≤ 0.001 and a log fold change of 2 indicating significance. To detect gene expression changes in darkness, pairwise comparison of the darkness treatment with the control light treatment were conducted.

To identify significantly enriched gene functions among the induced and repressed genes under darkness conditions, a GO enrichment analysis, as implemented in the trinity package (version 2.5.1), was performed. GO terms were classified with CateGOrizer^43^, using the EGAD2GO classification categories with the additional terms GO:0015979 “photosynthesis” and GO:0009579 “thylakoid”, applied for generating an overview of the GO enrichment analysis (Supplementary Fig. S2).

## Results and Discussion

### Reference transcriptome and comparative analysis

In this study, we present a comprehensive *de novo* reference transcriptome from *C. crenatum* under 18 different temperature and light regimes, covering initial, short-term and long-term acclimation. The Illumina Rapid Run yielded in ~29 million paired end reads with an average length of 300 bp, ~363 million single end reads with an average length of 100 bp, respectively. The *de novo* assembly yielded in 141,711 transcripts with an average length of 1,231 bp and an N50 of 1,592 bp. In total, 94.34% of the reads could be re-aligned (Bowtie2) to the assembly. Annotation of the assembled transcriptome against sprot BLASTX resulted in 58,826 hits (41.5%). The conducted KEGG mapping resulted in a high coverage, indicating that the established reference represents most parts of the functional genome (Supplementary Fig. S1).

BUSCO analysis using the dataset for plants (Embryophyta odb9) yielded in 39.1% complete, 4.2% fragmented and 56.7% missing BUSCOs, the reference set for Embryophyta odb9 only covers higher plants and orthologous groups that were described within these (n = 1,440). We additionally compared our reference to BUSCO plant v 1.1 (n = 956) and reached 67.2% complete, 10% fragmented and 22.8% missing BUSCOs, indicating a good coverage of orthologous groups^37^. For *Z. circumcarinatum* 76% complete BUSCOs, 8% fragmented and 16% were previously shown to be present accordingly^26^. BUSCO uses the single-copy characteristic of orthologues groups to estimate the contained redundancy of a (functional) genome assembly, but duplications and polyploidy may bias this function in Embryophyta^44^.

**Figure 1:**
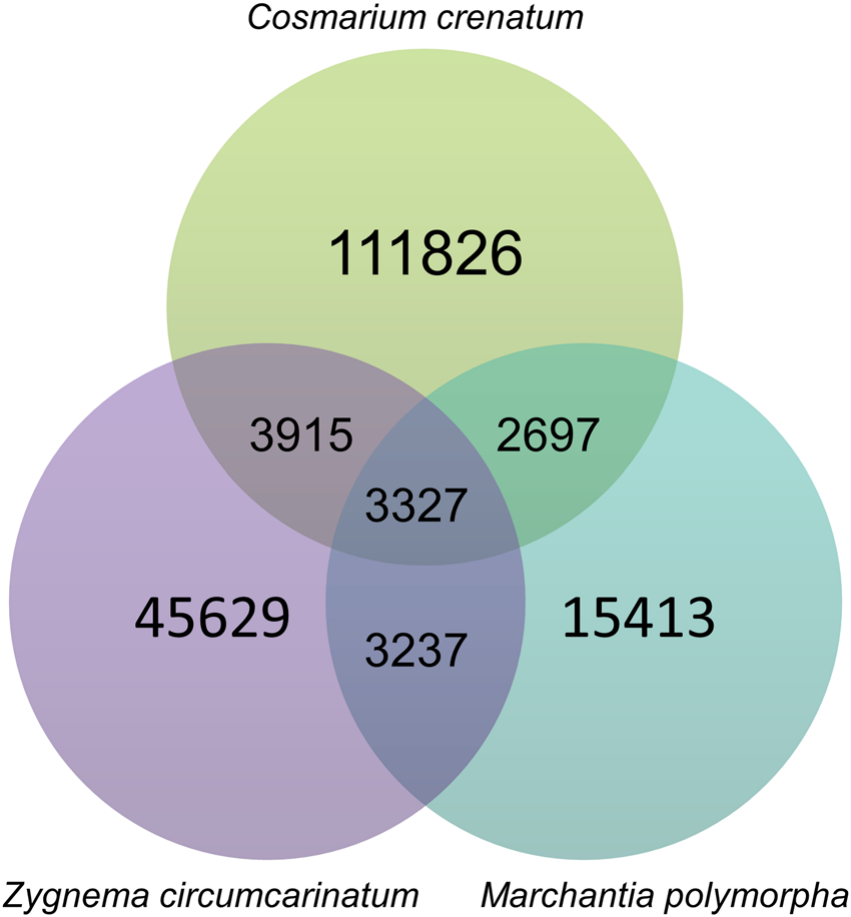
Venn diagram with reciprocal best hits between the reference transcriptomes of *Cosmarium crenatum* and *Zygnema circumcarinatum* and the genome of the liverwort *Marchantia polymorpha*. Larger numbers in circles indicate the number of unique protein sequences, shared sequences are found in overlapping areas. Protein sequences shared between all species tested are in the overlapping center.

The similarity of the Zygnematophyceae to early land plants is mirrored in the results from reciprocal BLAST analysis between *C. crenatum*, *Z. circumcarinatum*^26^ and *M. polymorpha*^41^ (Fig. 1). The three species share 3,327 protein sequences (center of diagram), the second highest number after direct comparison of the close relatives *C*. *crenatum* and *Z. circumcarinatum* with 3,915. *Z. circumcarinatum* and *M. polymorpha* share with 3,237 the second highest similarities. These results agree with Timme *et al*.^45^ who observed that *Spirogyra pratensis*, another member of Zygnematales, shares a high number of orthologous groups with land plants^45^.

More than 100 sequences in the comparison of *C. crenatum*, *Z. circumcarinatum* and *M. polymorpha* are encoding transcription factors, regulators or enhancers. An expansion of the number of transcription factors has been detected in the genome of *M. polymorpha*^41^. The expansion is hypothesized to may have allowed a diversification in the response to environmental changes^46^, which are more prominent and occur more rapidly on land than in an aquatic habitat. Along the shared sequences are plant hormone-responsive transcription factors, e.g. ethylene-responsive transcription factor (AIL1) and auxin response factors (ARFI). Phytohormones participate in a wide range of physiological and developmental processes and have been well studied in angiosperms^47^. For another streptophytic alga *S. pratensis* (Zygnematales) was recently shown that phytohormones alter the transcriptomic response of cell wall modification, photosynthesis and abiotic stress^27^. Our data indicates that phytohormones might also play a role in development and/or metabolism in *C. crenatum*. However, to confirm this hypothesis further studies should be conducted to investigate in more detail if and how phytohormones affect *C. crenatum*.

### Gene expression analysis of genes responding to darkness

Sequencing yielded in ∼14 million reads per replicate and on average 12 million reads were aligned to the reference (79%). In total, 7,905 differentially expressed genes (DEGs) were found, of which 3,767 transcripts were induced and 4,138 repressed in darkness. About 28% of the up-regulated and 47% of the down-regulated contigs could be successfully assigned an annotation. Furthermore, all differentially regulated genes were annotated using NCBI non-redundant (nr) database with diamond local aligner^48^ (cut-off 1E^−9^), which increased the number of annotated functions to about 40% in the up-regulated genes and 65% of the down-regulated genes. A full list of DEGs can be retrieved from the Supplemental Material (Supplemental Table 1).

### Functional annotation of darkness-responding transcripts

GO enrichment analysis resulted in 316 enriched GO terms among the in darkness up-regulated DEGs and 858 among the down-regulated transcripts in darkness (*p* < 0.01). General metabolism and carrier proteins were significantly enriched among both, the up- and down-regulated transcripts in darkness. Enriched GO terms among the down-regulated transcripts belonged mainly to the categories lipid metabolism, cofactor metabolism, energy/tricarboxylic acid (TCA) cycle and photosynthesis.

**Figure 2:**
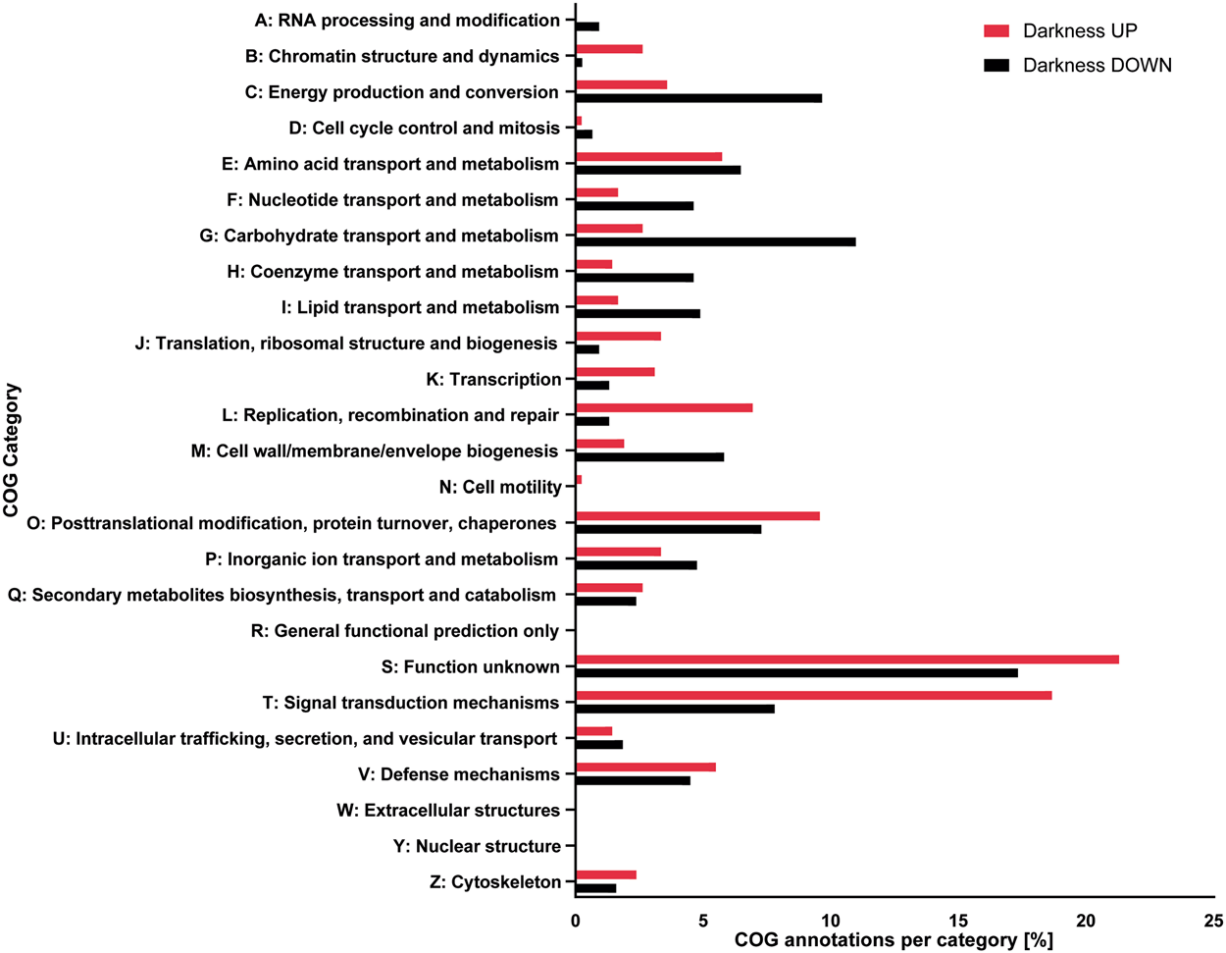
Categorized (A to Z) Clusters of Orthologous Groups (COG) analysis for darkness-induced and darkness-repressed transcripts, in percent (%) excluding “X: No COG/KOG” per group.

The distribution of annotated COG categories of regulated transcripts indicates a broad response to the darkness treatment regarding cellular functions. Largest percentages of DEGs were found in the category “unknown function” and “signal transduction mechanisms”. Darkness triggered more induction than repression of transcripts in several categories, e.g. “chromatin structure and dynamics”, “translation, ribosomal structure and biogenesis” and “signal transduction mechanisms”. Furthermore, darkness caused more down- then up regulation of transcripts belonging to the classes “energy production and conversion”, “carbohydrate transport and metabolism”. “Carbohydrate transport and metabolism” was the second largest number of COGs found in the down-regulated transcripts with 11.98%, followed by “energy production and conversion” with 9.66% (Fig. 2).

### Physiological response and gene expression adjustments to darkness

Under darkness cell density was roughly stable from day 1 (129,347 ± 1,063 cells mL^−1^) to day 7 (128,890 ± 4,336.15) with a decrease of 0.35%, indicating no cell divisions. Under low light cell density was highest at day 1 (114,803.75 ± 4,582.15), and decreased gradually until day 7 (94,662.5 ± 7,647.77) by 17.5%.

**Figure 3:**
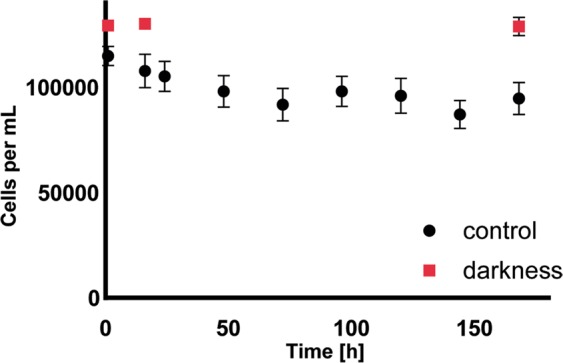
Cell number (cells/mL) of *C. crenatum* cultivated in 100 µmol photons m^−2^ s^−1^ (control) and darkness at 2 °C, error bars indicating standard deviation (n = 8).

After one week cell density remained the same in darkness (Friedman’s, *p* < 0.05, n = 8) (Fig. 3), this heterogeneous response is also reflected in the underlying molecular response in cell growth associated mRNAs. Expression of transcripts annotated endoglucanase 1 and 13 were regulated with logFC of 5.87 and −4.24, respectively (Table 1). Up- regulation was found for the probable pectinesterase 53 (logFC of 7.04), down-regulation was observed for pectinesterase 31 (logFC −4.42). Transcripts involved in cell wall growth, like xyloglucan endotransglucosylase/hydrolase and transcripts coding for various expansins, were repressed with logFC from −2.8 to −6.9. Expansins are proteins with diverse classification that induce loosening of wall molecules and subsequently cell extensibility^49^. Although often theorized about an enzymatic function; so far no catalytic activity has been determined^50^. In *Micrasterias denticulata* (Desmidiales), four different expansins are transcriptionally active during reorganization, assembly and selective degradation of the cell wall, two of the genes were active in earlier morphogenetic stages and two being induced in later stages^51^. Based on these findings, a manual BLASTx analysis was performed and the sequences of transcripts DN11092_c0_g1, DN11092_c0_g2, DN13260_c0_g1 and DN19416_c0_g1 retrieved as best hits the expansins of *M. denticulata*, which were all down-regulated in the darkness dataset. Furthermore, the involvement of xyloglucan endotransglucosylase/hydrolase (XTH) in cell wall loosening, strengthening and therefore growth is suggested for desmids^51^. All regulated transcripts encoding XTHs of *Cosmarium* were manually analyzed in BLASTx and corresponded to the XTH of *M. denticulata*, except for transcript DN18252_c0_g1, which retrieved a predicted XTH in *Brassica rapa*. All XTH-encoding genes were down-regulated in darkness, except for the one with the *B. rapa* annotation. These expression patterns are in accordance with the transcriptomic processes of *M. denticulata* as described by Vannerum *et al*.^51^, where the four expansin genes and the XTH were only significantly regulated during growth or in the initiation of it. Generally, when cells are in a growing stage one or more expansin genes are involved^49^. Our findings in the gene expression of expansins and XTHs in combination with no significant changes in cell counts in *Cosmarium*, indicate that neither cell divisions nor substantial modification of the cell wall occurred in darkness.

**Table 1:** Selected list of differentially expressed transcripts in response to darkness (complete list in Supplementary Table S1).

Contig ID	Putative gene product	E-value	logFC
**Cell wall development**			
DN15769c0g1	Endoglucanase 1	9.67E-126	5.87
DN19491c0g1	Endoglucanase 13	1.62E-63	−4.24
DN21391c0g1	Pectinesterase 31	1.79E-50	−4.42
DN14496c0g1	Putative xyloglucan endotransglucosylase/hydrolase protein 1	3.03E-23	−6.80
DN11092c0g1	Expansin-A8	2.93E-15	−6.92
DN13260c0g1	Expansin-A18	9.92E-29	−2.87
DN19416c0g1	Expansin-A14	8.91E-25	−5.64
DN14554c0g1	Probable xyloglucan endotransglucosylase/hydrolase protein 18	2.36E-16	−4.93
DN18208c0g2	Probable xyloglucan endotransglucosylase/hydrolase protein 33	4.61E-25	−4.05
DN18252c0g1	Xyloglucan endotransglucosylase/hydrolase protein 31	5.15E-10	2.03
**Photosynthesis, photorespiration and accessory pigments**			
DN16759c3g1	Chlorophyll a-b binding protein	3.66E-94	−10.05
DN13506c0g2	Photosystem I reaction center subunit II	3.3E-89	−4.00
DN10003c0g1	Photosystem II 10 kDa polypeptide	2.18E-22	−2.79
DN10098c0g1	Plastocyanin	6.83E-47	−5.03
DN11429c0g1	Cytochrome b6-f complex iron-sulfur subunit 2	3.65E-65	−3.43
DN22938c0g3	Cytochrome c6	8.24E-31	−2.13
DN22290c0g6	Ribulose bisphosphate carboxylase large chain	0	−2.32
DN12878c0g1	Ribulose bisphosphate carboxylase small chain C	6.32E-46	−2.60
DN16824c1g1	Glyceraldehyde-3-phosphate dehydrogenase GAPB	0	−3.39
DN16824c1g2	Glyceraldehyde-3-phosphate dehydrogenase A	0	−4.34
DN19513c0g2	Phosphoribulokinase	0	−5.48
DN19513c0g1	Phosphoribulokinase	3.64E-32	−5.01
DN16537c0g1	Tetrapyrrole-binding protein,	5.68E-37	−5.12
DN23145c0g1	Chlorophyll synthase	2.06E-144	−2.71
DN19195c1g2	Magnesium protoporphyrin IX methyltransferase	1.63E-99	−5.95
DN21095c1g2	Pheophytinase	3.79E-17	2.20
DN14118c0g1	Electron transfer flavoprotein subunit beta	8.92E-108	2.03
DN9942c0g1	Chlorophyllase-1	1.43E-32	2.42
DN21727c0g1	Violaxanthin de-epoxidase	2.88E-127	−2.18
DN23964c2g2	Zeaxanthin epoxidase	8.8E-61	2.05
DN16052c1g2	ATP-dependent 6-phosphofructokinase 4	3.77E-49	−6.82
DN20695c0g1	Cytochrome c	3.3E-63	−7.01
DN15902c0g3	NADH dehydrogenase 1 alpha subcomplex subunit 8-A	8.93E-35	−3.48
DN13480c1g15	Cytochrome b	1.01E-24	−2.05
DN22923c1g1	ATP synthase subunit beta	0	−2.05
DN15210c0g2	ATP synthase subunit epsilon	3.5E-19	−2.04
**Carbohydrate metabolism and associated transport**			
DN21236c0g2	Sucrose synthase 4	1.18E-71	2.88
DN24074c1g2	Probable starch synthase 4	2.34E-119	2.81
DN21996c0g1	Beta-amylase 3	3.04E-153	2.47
DN19743c0g2	Beta-amylase 7	8.54E-18	2.20
DN23791c2g1	Soluble starch synthase 3	4.15E-17	3.43
DN22147c0g1	Beta-amylase	3.13E-109	2.07
DN23709c2g1	Sugar transport protein 9	4.14E-75	6.04
DN23709c1g1	Sugar transport protein 7	1.12E-80	4.25
DN23709c2g3	Sugar transport protein 13	3.41E-93	2.26
**Lipid metabolism**			
DN21626c0g2	Delta(12)-acyl-lipid-desaturase	1.33E-139	−10.36
DN23736c0g1	Oleoyl-acyl carrier protein thioesterase	3.87E-60	−6.53
DN16200c0g1	Acetyl-CoA carboxylase carboxyl transferase subunit alpha	1.29E-72	−3.84
DN24419c0g2	Acetyl-CoA carboxylase 2	4.97E-25	−3.77
DN21155c3g1	Phospholipase SGR2	3.55E-119	4.20
DN21919c0g1	Triacylglycerol lipase 1	1.21E-30	4.83
DN13622c0g1	Autophagy-related protein 9	1.08E-10	2.04
DN15846c2g2	Autophagy-related protein 11	4.84E-10	2.07
DN16399c0g1	Autophagy-related protein 22-1	3.5E-21	2.37
**Epigenetic modifications**			
DN23893c0g2	Histone-lysine N-methyltransferase ATXR3	8.39E-114	2.19
DN19070c1g3	Histone-lysine N-methyltransferase family member SUVH2	8.14E-26	3.62
DN14298c1g2	Lysine-specific histone demethylase 1B	2.87E-24	3.16
DN20928c2g1	Histone acetyltransferase GCN5	3.45E-77	2.38
DN11922c0g1	Histone-lysine N-methyltransferase SUVR5	2.77E-11	2.45
DN26242c0g1	Histone-lysine N-methyltransferase SUVR4	5.96E-12	3.37
DN19418c0g3	Histone acetyltransferase GCN5	7.60E-11	2.57

**Figure 4:**
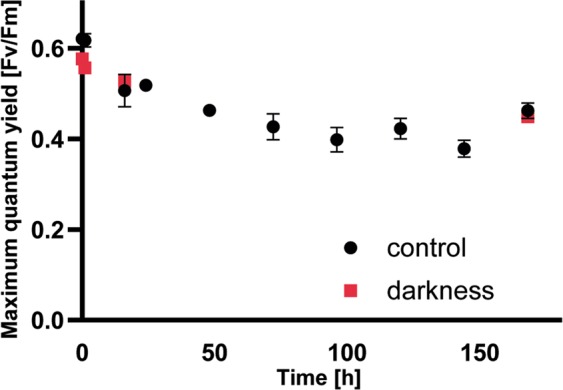
Photosynthetic efficiency of PSII (Fv/Fm) of *C. crenatum* cultivated in 100 µmol photons m^−2^ s^−1^ (control) and darkness at 2 °C, error bars indicating standard deviation (n = 8).

A significant difference in the photosynthetic response was observed between groups in darkness (D) and control conditions (C) for measurement point zero and after one hour (Friedman’s, *p* ≤ 0.05, n = 8). After one week of experiment, Fv/Fm was 0.449 (±0.008) in D and 0.461 (±0.016) in C (Fig. 4).

A slight decrease of Fv/Fm was overall observed and had been detected for several *Cosmarium* strains at low temperatures before^13^. Our results with 0.449 (78% of t0) in darkness and 0.461 (74% of t0) at 100 µmol photons m^−2^ s^−1^ are matching the findings from Stamenkovic and Hanelt (2013), where the Fv/Fm in *C. crenatum* has decreased to 0.5 after two weeks at 0.6 °C and 30 µmol photons m^−2^ s^−1^. A similar response was observed from *Micrasterias denticulata*, where after nine weeks of darkness the Fv/Fm dropped to 76% of t0^52^. Furthermore, it has been shown that cold temperatures reduce the activity of the Calvin cycle, which leads to a decreased oxidation of electron acceptors (e.g. NADPH) and even low light intensities may damage the photosynthetic apparatus by forming reactive oxygen species (ROS)^53^. As photoinhibition caused by cold shock, chilling stress and the resulting formation of ROS^54^ are unlikely to occur under prolonged darkness, other mechanisms, e.g. chlororespiration, might be responsible for the reduction of Fv/Fm. A slight decrease in Fv/Fm after the short-term exposure to darkness had been described for the Antarctic alga *Palmaria decipiens* (Rhodophyta)^55^, but these authors concluded that the further reduction of the Fv/Fm was a sign for the start of the degradation of the photosynthetic apparatus and do not provide a theory for the decrease in the first place. The mechanism of chlororespiration has been theorized as an alternative respiratory chain within the thylakoid membrane, enabling electron transport even in the dark^56^. In theory, the plastoquinone (PQ) pool is oxidized in darkness as light energy is not provided for electron delivery by the oxygen-evolving complex, reaction center II and electron transport to PS I^56^. In reality, there are indications that parts of the PQ pool remain or become reduced also in darkness e.g. in tomato after 5 hours of darkness^57^ and in the green alga *Chlamydomonas reinhardtii*^58^ causing a decrease of the maximal possible value of Fv/Fm. The specific pattern of a reduced Fv/Fm after one week in darkness exhibited by *C. crenatum* may reflect a specific adaption to unfavorable conditions of this Arctic opportunist. There are at least six reactions occurring within the chloroplast that can lead to a reduced plastoquinone pool^59^. A central oxidase located in the thylakoid membrane, the plastid terminal oxidase (PTOX also called IMMUTANS or AOX4), may participate in the electron transfer in the light-independent chlororespiration and already shown to be the major oxidase involved in the process in *C. reinhardtii*^58^. Although not significantly regulated in darkness in *C. crenatum*, there is at least one copy of the gene present and therefore an indication of occurrence of an alternative respiratory mechanism within the chloroplast. Additionally, a significant alteration of the gene expression of photosynthetic components and associated pathways (e.g. chlorophyll metabolism) was shown for *C. crenatum*. These hidden alterations, not detectable by the Fv/Fm parameter, were observed in the brown macroalga *Saccharina latissima*^60,61^. In our experiments, *C. crenatum* responded to darkness with a down-regulation of nearly all transcripts coding for components of the photosynthetic apparatus. Mainly all transcripts encoding proteins of PSI and PSII were down-regulated, the strongest repression was observed for a chlorophyll a-b binding protein (logFC −10.1). Genes encoding parts of the electron transport chain like cytochrome b6-f complex proteins, plastocyanin and cytochrome c6 were repressed with fold changes of −3.44, −5.0 and −2.1, respectively. The mRNA level of most of the photosynthesis associated genes was reduced in darkness, which is in accordance with findings in *A. thaliana* for light-controlled metabolic and regulatory pathways^62^. Furthermore, genes involved in photosynthesis were down-regulated in the Antarctic marine diatom *Fragilariopsis cylindrus* after one week of prolonged darkness compared to expression under continuous light^63^, indicating a common response.

Genes associated with photosynthetic carbon fixation of the reductive pentose phosphate pathway were also down-regulated. Transcripts encoding ribulose bisphosphate carboxylase (RuBisCO) small and large chains were repressed with −2.6 and −2.3. Two chloroplast localized phosphoribulokinase (PRK) transcripts were down-regulated with logFC of −5.0 and −5.5. The glyceraldehyde-3-phosphate dehydrogenase (GAPDH) was repressed with logFC −4.3 and −3.4. Both are essential enzymes in carbon fixation and glycolysis. The two subunits of GAPDH with chloroplast localization, GAPA and GAPB, were repressed in darkness. The GAPB subunit originated from duplication of the GAPA subunit, but differs in 20% of its amino acids from GAPA^64^. The gene fusion leading to the higher plant chloroplast-specific GAPDH complex took place in the time before the transition from water to land habitat^65^ and therefore GAPB is found in nearly all groups of Charophyta^66^. The occurrence of both subunits in *C. crenatum* supports the hypothesis that the gene fusion happened before entering terrestrial habitats. The small Calvin cycle protein (CP12) is known for its regulatory interaction with the photosynthetic GAPDH^67^. It forms disulfide bonds with the PRK in darkness and is directly lowering enzyme activity^68^, therefore enabling metabolic flexibility in carbon fixation, which happens upon irradiation. This is in accordance with the gene expression of *C. crenatum* as GAPA, GAPB and PRK were all significantly repressed. CP12 is not significantly regulated in darkness in *C. crenatum*, which is different from A. thaliana, where after five days still an up-regulation of expression was observed. Nevertheless, the data indicates a reduction of the other gene products associated with the Calvin cycle and a reduced carbon fixation in darkness.

### Chlorophyll and carotenoid metabolism

Transcripts involved in chlorophyll or porphyrin biosynthesis like tetrapyrrole-binding protein (GUN4C), chlorophyll synthase and magnesium protoporphyrin IX methyltransferase were down-regulated with logFC of −5.1, −2.7 and −5.9, respectively. Transcripts coding for proteins involved in chlorophyll catabolism, e.g. pheophytinase, chlorophyllase-1 and electron transfer flavoprotein subunit beta (ETFB or ETF β) were up-regulated resulting in logFCs of 2.2, 2.4 and 2.0. The electron transfer flavoprotein (ETF)/ETF ubiquinone oxide reductase (ETFQO) electron transfer complex is essential for *A. thaliana* under extended darkness conditions, and is supposed to be involved in the chlorophyll degradation pathway activated during dark-induced carbohydrate deprivation and catabolism of leucine^69^. Even though the expression of ETF β, is not affected in response to prolonged darkness in *A. thaliana*, it has been shown that the ETF re-oxidizing protein ETFQO responds with high expression levels in sucrose-starved conditions in darkness^70^. As structure and function of ETF and ETFQO are well conserved it can be concluded that *C. crenatum* did not enter a sugar starvation phase as the expression of ETFQO was not significantly regulated. Adding up to this, the isovaleryl-CoA dehydrogenase (IDV) gene has been shown to be active under sucrose starvation in almost all cell types of *A. thaliana*^71^ and is present in the here presented reference transcriptome (DN22252_c1_g1_i5, E:0), but not significantly regulated in darkness.

Central enzymes of the xanthophyll cycle were significantly differentially expressed in darkness. Violaxanthin de-epoxidase showed lower mRNA level in darkness (logFC −2.2) whereas transcripts encoding zeaxanthin epoxidase were induced (logFC of 2.1). In plants and green algae the violaxanthin-cycle is present and serves as a control mechanism against the formation of ROS^72^. The onset of violaxanthin de-epoxidase under high light as well as contrary the activation of zeaxanthin epoxidase in darkness is an early defined system^73^ and in accordance to our findings. For *C. crenatum* it has been shown that the xanthophyll cycle pool strongly reacts to high light with an accumulation of the intermediate antheraxanthin, but remains stable in low light and recovery^74^. Therefore, the here detected expression of genes of the xanthophyll cycle is in accordance with previously published physiological data of this isolate.

### Energy metabolism

Exposure to long-term darkness caused repression of most transcripts involved in primary energy metabolism, e.g. glycolysis or tricarboxylic acid cycle (TCA). Mainly all key components of glycolysis were repressed, starting with the ATP-dependent 6-phosphofructokinase (logFC −6.8) (Table 1). Furthermore, expression of transcripts also coding for enzymes and membrane complexes of the oxidative phosphorylation, like NADH dehydrogenase to cytochrome c, cytochrome b and the subunits beta and epsilon of ATP synthase, were down-regulated with logFCs between −2.05 and −7.0. Contrarily, synthesis of numerous transcripts that are encoding genes of the starch metabolism, like starch synthase; sucrose synthase and beta-amylase were induced after one week of darkness with logFCs between 2.0 and 3.4. Another indication for the use of organic molecules are the expression patterns in carbohydrate transporters e.g. specific sucrose transporters with logFC of 2.9 and hexose transporters with an increase of up to 6-fold and can directly be linked to energy metabolism. Starch is the most abundant carbohydrate storage molecule in land plants and organized in pyrenoids within the chloroplast^7^, if present. In general, starch is stored during the day inside the chloroplast of plants and metabolized at night^75^. In the green microalga *Acutodesmus obliquus* it has been recently proven that starch acts as the major transitory energy storage compound, which was accumulated in the very last part of the illuminated period and consumed in the whole cause of darkness^76^. For *A. thaliana* it has been confirmed on the protein level that when facing very short light periods or extended darkness, starch synthesis rate increases during the day and degradation rate decreases rapidly during night^77^. Furthermore, the content of sugars and amino acids in the end of the dark period were largely independent from the length of the radiation period, presumably plants acclimated quickly to the prolonged dark conditions^77^. Light regulates transcript abundance of a large number of genes^2^ and darkness appears to be a trigger *vice versa*. The exposure of *A. thaliana* plants to altered conditions of light availability leads to an adjustment of transcript level of starch degradation-related enzymes^75^. In continuous darkness, starch degradation-related gene transcripts decline within the first 9 h and are barely expressed in the experiments after 9 h of darkness. This is in contrast to the results found in the polar *C. crenatum* where expression of transcripts of genes encoding beta-amylases (AMYB, BAM3, BAM7) as well as different starch synthases (Soluble starch synthase 3 + 4) and sugar transporters were significantly induced even after one week of darkness. Especially, beta-amylase (BAM) is required for standard starch breakdown in *A. thaliana*^78,79^. From the patterns in energy metabolism it is assumed that *C. crenatum* survives extended darkness periods by the breakdown of starch stored within the chloroplast.

In our dataset the mRNA levels of specific sugar transport proteins 7, 9 and 13 were induced in darkness (log FC 4.2, 6.0 and 2.2), which are localized within the plasma membrane and mediate active uptake of hexoses e.g. glucose^80^. There are indications that *C. crenatum* may survive the extended dark period due to heterotrophic utilisation of hexoses derived from the very specific nature of desmids. Desmidiaceae produce a sheath of extracellular polymeric substances (EPS) which are known to function in various matters e.g. movement or adhesion^81^ and nutrient capture^82^. The EPS cover of *Cosmarium turpinii* has been shown to mainly consist of xylose, galactose, glucose and other organic carbohydrates^83^. Therefore it may be possible to utilize the monosaccharides in the EPS, but further investigations in heterotrophic conditions will be necessary in the future that monitor the uptake of hexoses from the sheath layer. It has been proven for many microalgae lineages to be capable of heterotrophic utilization of different carbon sources^84^, with most records from the green algae lineage. The ability to increase the heterotrophic potential and the uptake of dissolved organic material or lipids has been reported from polar diatoms and may be a way to thrive within polar night conditions^85^.

Transcripts annotated with functions in the lipid metabolism were differently regulated in darkness, even though the number of repressed gene transcripts was higher. Strongest repressed gene with a logFC of −10.3 was a delta-(12)-acyl-lipid-desaturase, but amongst the other repressed transcripts many different desaturases were present (Table 1). Transcripts involved in key steps of lipid biogenesis like acetyl-coenzyme A carboxylase (logFC −2.4 and −3.8) and oleoyl-acyl carrier protein thioesterase (log FC −6.5) were also repressed. The phospholipase SGR2 was induced in darkness *in C. crenatum* with logFC of 4.2. It is known to be involved in gravitropism in *A. thaliana* and induces leaf movement in darkness^86^. Therefore, this is an indication of the gene for darkness-specific response, already present early in the Zygnematophyceae. Specific mRNAs of genes encoding long-chain-alcohol-oxidases, like lipase (LIP1) were induced under darkness with logFC 4.8. These are involved in triacylglycerol (TAG) breakdown^87^ and may be a source of NAD(P)H when cells are low in major carbon reserves like starch^59^. Recently it has been indicated for a close relative of *C. crenatum*, *Micrasterias denticulata*, that classical autophagy and lipid degradation appears during carbon starvation^52^. In *M. denticulata* during carbon starvation (e.g. induced by darkness) lipid droplets are transported outside the chloroplast, where they occasionally fuse with the vacuolar compartments and are degraded. Similar shifts of chloroplast lipids to the cytoplasm has been observed in another transmission electron microscope (TEM) study in starved cells of the green alga *Chlorella*^88^. This accumulation of lipid bodies by microalgae is a response to store energy in unfavorable environmental conditions, hypothesized to quickly utilize TAG to synthetize metabolites or biomembranes once conditions resume to normal^89^. Classical autophagy of lipids (or lipophagy) has been indicated for *M. denticulata* and also an orthologue of the autophaphy-related gene 8 was shown to be present in it^52^. At least one ATG8 orthologue is present in *C. crenatum* in our reference, but not significantly regulated. In darkness, different autophagy-related genes (ATG9, ATG11, ATG22-1) are up-regulated and can serve for autophagy-induced lipid mobilization or recycling. Therefore, it may be assumed that storage lipids and TAGs are another possible energy source utilized in *C. crenatum* in darkness.

### Epigenetic modifications

We observed enhanced mRNA levels in response to darkness for several transcripts correlated to histone modifications. Histone modifications influence chromatin structure and gene activity by changing DNA–histone interaction and accessibility of transcription factors^90^. Transcripts encoding proteins involved in H3 K4 methylation/demethylation processes were induced under darkness, e.g. histone-lysine N-methyltransferase ATXR3 (logFC 2.2), histone-lysine N-methyltransferase family member SUVH2 (logFC 3.6) and lysine-specific histone demethylase 1B (logFC 3.2). Genes connected to methylation of H3 K9 showed significantly higher transcript abundances, i.e. histone-lysine N-methyltransferase SUVR4 (logFC 3.4) and SUVR5 (logFC 2.5). Additional, we observed induction of 2 transcripts involved in histone acetylation, namely histone acetyltransferase GCN5 and increased DNA methylation, which were up-regulated with log FC 2.4 and 2.6. Even though the presence of histone methyltransferases in algae have been reported^91^ up to now little is known on changes in histone modification status under abiotic stresses in algae. A study conducted on the unicellular alga *C. reinhardtii* showed that copper starvation and heat stress causes histone acetylation of H3/4 as well as changes in the methylation state of H3 K4^92^. Histone modifications are involved in plant stress response, hypoxia in rice seedlings caused dynamic and reversible changes of histone H3 K4 methylation and H3 acetylation^93^. Heat stress response in *A. thaliana* causes enrichment of H3 K4 trimethylation and H3 K9 acetylation^90^. Furthermore, it was observed that dehydration stress in *A. thaliana* triggers dynamic changes in genome-wide histone H3K4 methylation patterns^94^. Our results indicate that adaptation/acclimation to darkness in *C. crenatum* involves a sophisticated network of histone modifications. In general, H3 K4 and H3 K36 methylation are correlated with gene activation, whereas H3K9 and H3K27 methylation as well as H3 acetylation are associated with gene silencing^95^. However, as the same histone mark can have different functions in different organisms^96^, more detailed studies should focus on how gene expression adjustments in response to abiotic changes in algae are associated with certain histone modification. As a recent study on microevolution in *C. reinhardtii* showed that transgenerational epigenetic effects play an important role in adaptive evolution^97^, future studies on histone modifications in *C. crenatum* should investigate whether these are passed through mitotic cell division.

### Ecological implications and darkness acclimation

In our experiments, the switch to absence of light was the required trigger for expression changes in the transcriptome and acclimation to winter conditions. According to a communication by Mix (1970), *Cosmarium* species have developed gelatinous sheaths outside the cells or incorporate storage molecules, especially starch and lipids, to protect the vegetative cells from damage caused by freezing^98^. The mucilage sheaths also have an influence on the sinking rate, which ensures that certain species (e.g. *C. botrytis*) rapidly sink to the ground in a water body^99^. Usually freezing damage does not occur to benthic *Cosmarium* species in ponds and lakes in the high Arctic as, depending on depth, water bodies do not freeze solid to the ground (e.g. Greenland with lowest temperature between 0.01–0.1 °C in 2 m depth)^100^. In accordance with our findings, laboratory experiments on *C. botrytis* showed a survival of vegetative cells in darkness^101^.

*C. crenatum* maintains a vegetative cell which is non-dormant and holds the ability to photosynthesize when exposed to light. Polar winter laboratory experiments on the endemic Antarctic red alga *Iridaea cordata* over six months of darkness show a similar pattern^102^. The maximum photosynthetic rate is slightly decreased within the first months, but afterwards maintained at a certain level. *I. cordata* uses floridean starch as energy source in darkness and maintains the structure of the photosynthetic apparatus without structural changes. As *C. crenatum* is exhibiting gene expression associated with starch catabolism but no growth occurs it can be hypothesized that the species has a comparable survival strategy. Further experiments with longer darkness periods will be necessary to verify its ecological adaption to polar winter.

## Conclusion

It is demonstrated that darkness was associated with down-regulation of genes of the primary metabolism, photosynthesis as well as cell divisions and photosynthetic efficiency in *C. crenatum* as ecophysiological acclimation to enhanced dark periods. As gene expression of transcripts encoding starch catabolism and to some extent sugar transport proteins is clearly induced in darkness, we assume that these pathways ensure main energy supply in dark periods, and therefore phases of heterotrophic energy supply when photosynthesis is not efficient or does not occur at all. Chlororespiration may play a role in electron transport within the chloroplast, but needs to be further investigated. We were able to show that cell division stops immediately and after one week of darkness no significant changes are detectable in the number of cells, resulting in a mechanism to save storage molecules. Significant changes in gene expression were observed under conditions where the Fv/Fm remained unchanged. Moreover, genes of the primary energy metabolism which involve glycolysis, TCA and oxidative phosphorylation are repressed. During a long dark period under polar conditions it could be beneficial for this organism to avoid extensive usage of storage compounds. Considering the polar origin of *C. crenatum* MZCH 561, its tolerance to low temperatures in combination with total darkness was shown as the algae were able to cope with the conditions and specifically react. A period of prolonged darkness (up to 9 months) and low temperatures seem to be key altering factor in this environment during polar winter.

Artificial laboratory conditions are hardly mimicking the exact conditions of a long polar winter where gradual acclimation in response to seasonal change is acquired. Therefore, future studies should include year-round *in situ* sampling in the environment to further shed light on dark acclimation and recovery of these photoautotrophs.

## Supplementary information

Supplementary Figures

Supplementary Table

## Data Availability

The cleaned raw sequencing data are deposited in the European Nucleotide Archive (ENA) at the European Molecular Biological Laboratory–European Bioinformatics Institute under study accession number PRJEB30351 https://www.ebi.ac.uk/ena/data/view/PRJEB30351.
