# Supplementary material for: Darkness-induced effects on gene expression in Cosmarium crenatum (Zygnematophyceae) from a polar habitat
Source: Sci Rep. 2019 Jul 22;9:10559. doi: 10.1038/s41598-019-47041-7 (PMC6646379; doi:10.1038/s41598-019-47041-7)
Supplement: Supplementary file 1 — Supplementary Figures [file 41598_2019_47041_MOESM1_ESM.pdf]

Supplementary Figures and legends

Darkness-induced effects on gene expression in *Cosmarium crenatum* (Zygnematophyceae) from a polar habitat

Florian Mundt, Dieter Hanelt, Lars Harms, Sandra Heinrich

Supplementary Figure 1.

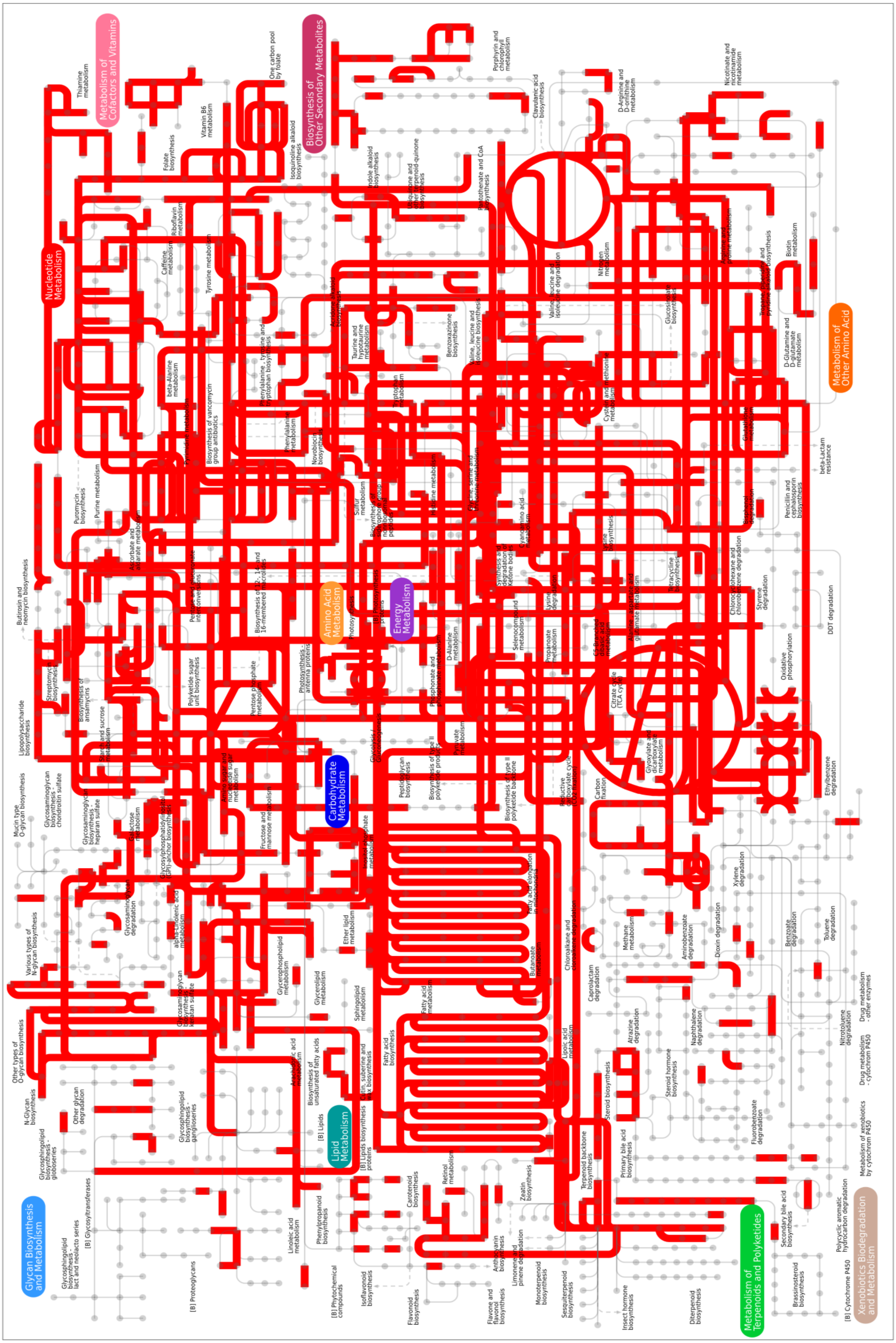

Supplementary Figure 2.

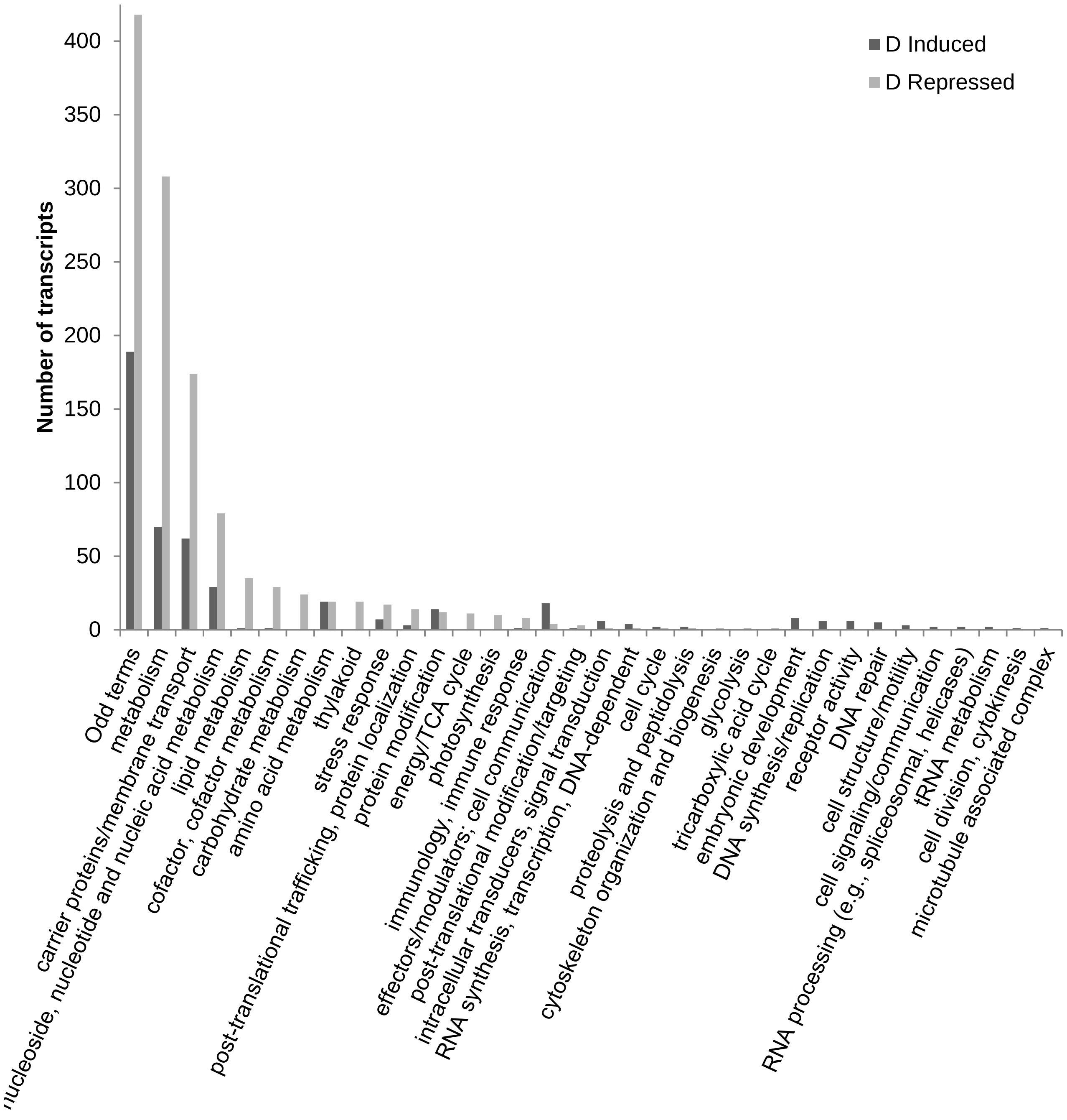

Supplementary Figure legends

Supplementary Figure 1. Kyoto Encyclopedia of Genes and Genomes (KEGG)[1,2,3] metabolic pathway mapping of orthology (KO) and clusters of orthologous groups (COG)

Supplementary Figure 2. Gene ontology enrichment analysis results from induced (D Induced) and repressed (D Repressed) genes in darkness conditions. Sorting performed with CateGOrizer using standard „egad“ ancestor classification categories adding GO:0015979 “photosynthesis” and GO:0009579 “thylakoid” (p<0.001)

References

1 Kanehisa, M. & Goto, S. KEGG: Kyoto Encyclopedia of Genes and Genomes. Nucleic Acids Res **28**, 27-30, doi:Doi 10.1093/Nar/28.1.27 (2000).

2 Kanehisa, M., Furumichi, M., Tanabe, M., Sato, Y. & Morishima, K. KEGG: new perspectives on genomes, pathways, diseases and drugs. Nucleic Acids Res **45**, D353-D361, doi:10.1093/nar/gkw1092 (2017).

3 Kanehisa, M., Sato, Y., Furumichi, M., Morishima, K. & Tanabe, M. New approach for understanding genome variations in KEGG. Nucleic Acids Res **47**, D590-D595, doi:10.1093/nar/gky962 (2019).
